# Efficacy and tolerability of repository corticotropin injection in patients with persistently active SLE: results of a phase 4, randomised, controlled pilot study

**DOI:** 10.1136/lupus-2016-000180

**Published:** 2016-10-21

**Authors:** Richard Furie, Margaret Mitrane, Enxu Zhao, Maya Das, Daner Li, Patrice M Becker

**Affiliations:** 1 Hofstra Northwell School of Medicine, Northwell Health, Great Neck, New York, USA; 2 Manhattan BioPharm Consultants LLC, New York, New York, USA; 3 Research & Development, Mallinckrodt Pharmaceuticals Inc., Ellicott City, Maryland, USA

**Keywords:** Autoimmune Diseases, Corticosteroids, Systemic Lupus Erythematosus, Disease Activity

## Abstract

**Objective:**

To evaluate the efficacy of a prolonged-release formulation of a porcine adrenocorticotropic hormone analogue (repository corticotropin injection (RCI)) added to standard of care in patients requiring moderate-dose corticosteroids for symptomatic SLE.

**Methods:**

This prospective, randomised, double-blind, phase 4, pilot study (NCT01753401) enrolled 38 patients with persistently active SLE involving skin and/or joints. Enrolled patients received RCI, 40 U daily or 80 U every other day, or volume-matched placebo gel, for 8 weeks, with dose tapering to twice weekly during weeks 5–8. Efficacy endpoints included proportion of responders at week 4 based on a novel composite measure that included resolution of rash or arthritis measured using the hybrid SLE Disease Activity Index (hSLEDAI) without worsening British Isles Lupus Assessment Group (BILAG) scores in other organ systems at week 4 (primary), as well as improvements in total hSLEDAI and BILAG scores and other measures of skin and joint disease activity over the 8-week treatment period.

**Results:**

Response, as defined for the primary endpoint, did not differ significantly between the combined placebo and RCI-treated groups at week 4. At week 8, the proportion of responders was higher in RCI-treated patients but did not statistically differ between groups (RCI 40 U (53.8%), RCI 80 U (33.3%), combined placebo (27.3%)). However, RCI treatment was associated with statistically significant improvements in several secondary endpoints, including total hSLEDAI, total BILAG and Cutaneous Lupus Erythematosus Disease Area and Severity Index Activity scores within 8 weeks. Treatment was well tolerated.

**Conclusions:**

Although the primary endpoint was not met in this pilot study, secondary and post hoc analyses suggested that RCI was associated with improvements in SLE disease activity in a select patient population with steroid-dependent persistent disease.

**Trial registration number:**

NCT01753401; results.

**Key messages**

- This phase 4, 8-week, prospective, randomised, double-blind, placebo-controlled, pilot study yielded controlled evidence to evaluate the use of repository corticotropin injection (RCI; Acthar Gel) as a potential treatment option for patients with persistently active SLE involving skin and/or joints despite moderate-dose corticosteroid therapy.
- The study did not meet its primary endpoint (a novel composite responder index assessed at week 4), but improvements were seen with RCI versus placebo in standard outcome measures such as change from baseline in total hybrid SLE Disease Activity Index, and total British Isles Lupus Assessment Group score, by weeks 6–8.
- The overall incidence of adverse events was comparable in the RCI and placebo groups, and there were no unexpected adverse events reported.

## Introduction

SLE is a chronic, autoimmune disease characterised by the production of autoantibodies, immune complexes and proinflammatory cytokines that cause inflammation and tissue damage in various organ systems.^1^ Although diagnostic and therapeutic advances have improved 5-year to 10-year survival rates to more than 90%,^2^ ^3^ there remain significant unmet needs in the management of SLE, particularly among patients with persistent, treatment-refractory disease.^4^ Furthermore, current treatments have limitations.^5^

While some of the effects of adrenocorticotropic hormone (ACTH) to modulate inflammation are likely mediated by its ability to stimulate cortisol production by the adrenal gland, additional data suggest that direct effects of ACTH and other melanocortin peptides on cells of the immune system potentially contribute to its anti-inflammatory activity, independent of actions related to the hypothalamic–pituitary–adrenal axis.^6^ Preclinical studies suggest that melanocortin peptides, including ACTH and α-melanocyte-stimulating hormone (α-MSH), have diverse anti-inflammatory effects, including inhibition of leucocyte migration in organs and joints, decreased expression of proinflammatory cytokines and chemokines, and reductions in adhesion factor production and expression.^6^ ^7^ Melanocortin peptides may also have steroid-independent pro-resolving properties in inflammatory conditions, for example, by promoting efferocytosis, attenuating protease production by chondrocytes or altering the balance between osteoclast and osteoblast activity.^6^ The evolving data supporting steroid-independent properties of melanocortins have increased attention to these peptides as potential therapies for autoimmune conditions such as SLE.^6^ ^7^

Repository corticotropin injection (RCI; H.P. Acthar Gel, Mallinckrodt ARD, Ellicott City, Maryland, USA) contains a highly purified porcine ACTH analogue formulated to allow prolonged release after intramuscular or subcutaneous injection, and is approved by the US Food and Drug Administration for use during exacerbations and as maintenance therapy in selected patients with SLE.^8^ In a murine model of SLE, this preparation has been shown to reduce B-cell differentiation and development, and to decrease circulating autoantibodies, proteinuria, renal lymphocyte infiltration and glomerular immune complex deposition.^9^ Similarly, other investigators demonstrated that α-MSH attenuated manifestations of pristane-induced lupus in mice.^10^ Effects of RCI on human B-cell function were studied in vitro using peripheral blood B cells isolated from healthy human subjects after activation by interleukin 4 (IL-4) and CD40 ligand (CD40L). RCI dose dependently inhibited IL-4/CD40L-induced B-cell proliferation, expression of markers of immunoglobulin class switching and IgG production without enhancing cell death. These data suggest direct effects of RCI on human B-cell function and provide supportive evidence for steroid-independent effects of RCI when used as a treatment for SLE, an autoimmune disease characterised by B-cell activation and humoral autoimmunity.^11^

The use of ACTH preparations in SLE is supported by over 60 years of clinical experience,^12^ and a more recent open-label, single-arm case series involving 10 patients with chronic moderate-to-severe SLE, in which RCI resulted in significant improvements in SLE Disease Activity Index-2000 (SLEDAI-2K) scores and other measures of disease activity.^13^ Here, we report findings from a pilot 8-week, prospective, randomised, placebo-controlled study evaluating the efficacy and safety of RCI in patients with persistently active SLE despite moderate-dose corticosteroids.

## Methods

### Study design

This phase 4 pilot study (NCT01753401) consisted of an 8-week, randomised, double-blind, placebo-controlled treatment period followed by a 44-week open-label extension. The objectives of the double-blind phase were to investigate the effect of RCI on disease activity in SLE, and to assess safety. The study was conducted at 20 sites in the USA (see online supplementary file 1). The protocol was approved by the Institutional Review Boards at all participating centres, and the study was conducted according to the principles of the Declaration of Helsinki and Good Clinical Practice. All principal investigators and subinvestigators and study coordinators at each site were required to complete scale assessment training prior to enrolling any patients in the study. Because this was a pilot study, serial assessment done by a single examiner was not required. Data from the controlled phase of this pilot study are reported here.

10.1136/lupus-2016-000180.supp1 supplementary file

### Patients

Adult patients (age ≥18 years) were eligible to participate if they met at least four of the American College of Rheumatology revised diagnostic criteria for SLE^14^ and had active disease with arthritis and/or rash scored present on the hybrid SLE Disease Activity Index (hSLEDAI). Patients were also required to have a British Isles Lupus Assessment Group (BILAG)^15^ score of A or B in either the mucocutaneous or musculoskeletal domains. All patients were required to be seropositive for, or have a documented history of, ANAs and anti-dsDNA, anti-Smith or anti-cardiolipin antibodies. Persistent disease activity had to be evident despite the use of stable, moderate-dose corticosteroids (prednisone 7.5–30 mg/day, or equivalent) for at least 4 weeks prior to screening.

Principal exclusion criteria included initiation of corticosteroid treatment within 2 months prior to screening, active nephritis (defined as serum creatinine >221 µmol/L, protein:creatinine ratio >1.5 g/g or haemodialysis within 3 months prior to screening) and active central nervous system lupus requiring therapeutic intervention within 3 months before screening. Patients were excluded if they had received parenteral steroids within 1 month; oral steroids at doses >30 mg/day (prednisone or equivalent), ciclosporin or any non-biological investigational drug within 3 months; intravenous immunoglobulin or plasmapheresis within 4 months; cyclophosphamide within 6 months; or B-cell-targeted therapies, abatacept or any biological investigational agent within 12 months prior to screening. Other exclusion criteria included diabetes mellitus, pregnancy, known contraindications to RCI^8^ or clinically significant medical conditions that could compromise the patient's ability to complete the study. Written informed consent was obtained from all patients.

### Interventions and assessments

As RCI is volume dosed, patients were randomised in a 2:1:2:1 ratio to receive RCI 40 U or volume-matched placebo once daily (QD), or RCI 80 U or volume-matched placebo once every other day (QOD), by subcutaneous injection. During weeks 1–4, the dose volume could be reduced, corresponding to RCI doses of 16 U QD and 40 U QOD in the 40 U QD and 80 U QOD groups, respectively, if predefined safety criteria were identified. During weeks 5–8, the doses were tapered by a schedule provided in the protocol, reducing the frequency of administration such that all patients were receiving two doses of study medication per week by the end of the double-blind period. Concomitant corticosteroids, non-steroidal anti-inflammatory drugs, antimalarial agents, methotrexate, azathioprine and mycophenolate mofetil were permitted provided that eligibility requirements for these agents were met and the doses remained stable throughout the 8-week study period. Randomisation was performed using an interactive web response system. Both investigators and patients were blinded to study treatments. Placebo gel was identical to RCI in formulation but did not contain active drug.

Disease activity was assessed with the hSLEDAI^16^ and Physician's Global Assessment (PGA)^17^ at baseline and at weeks 2, 4, 6 and 8. In addition, the BILAG-2004, Cutaneous Lupus Erythematosus Disease Area and Severity Index (CLASI)^18^ and 28-joint count (using shoulder, elbow, wrist, knee, metacarpophalangeal and proximal interphalangeal joints)^19^ were assessed at baseline and weeks 4 and 8. Health-related quality of life (QoL) was assessed with the Medical Outcomes Survey Short Form-36 (SF-36)^20^ and the Krupp Fatigue Severity Scale (KFSS)^21^ at baseline and weeks 4 and 8. The SLE Responder Index (SRI)^22^ was calculated post hoc using data collected for the SLEDAI, BILAG and PGA at weeks 4 and 8. Serological markers of SLE disease activity (anti-dsDNA antibodies and complement C3 and C4) were also measured. Safety was assessed throughout the study by monitoring adverse events (AEs), physical examinations, measurements of vital signs and clinical laboratory investigations. All investigators were required to complete training on disease activity measures prior to study initiation.

### Outcome measures

The primary endpoint was a novel composite responder index defined as the proportion of patients responding to treatment at week 4, with response defined by a decrease in hSLEDAI score from 4 to 0 for arthritis, or from 2 to 0 for rash, with no worsening in other organ systems as assessed by BILAG.

Secondary endpoints included the proportion of responders at week 8, and changes in total hSLEDAI, BILAG-2004,^23^ CLASI, SF-36, KFSS or PGA scores from baseline at specific time points. The hSLEDAI is a modified version of the original SLEDAI and is identical to the SELENA-SLEDAI except for the scoring of proteinuria, which uses the SLEDAI-2K definition. Response to treatment on the basis of shifts in mucocutaneous and musculoskeletal BILAG scores (a shift from category A at baseline to category B, C or D, or from category B at baseline to category C or D) was assessed at weeks 4 and 8. Additional analyses performed post hoc included the proportion of patients who met the definition of an SRI response at weeks 4 and 8.

### Statistical analyses

No formal sample size calculation was performed for this pilot study, although it was anticipated that the results of this study would inform the sample size calculation for subsequent randomised trials of RCI in SLE. Data from placebo groups were pooled for reporting of results and statistical analyses. Because weekly RCI exposure was similar for the two RCI dosing regimens, statistical analyses were performed comparing pooled placebo data to specific RCI dose regimens, as well as to pooled RCI data. All efficacy and safety analyses were performed on a modified intention-to-treat (mITT) population, which included all randomised patients who received at least one dose of study medication and had at least one post-baseline assessment of efficacy or safety.

The primary endpoint (response rate at week 4), the response rate at week 8, and the SRI at both time points were analysed by means of an exact logistic regression model with treatment group (three levels: RCI 40 U or 80 U and combined placebo groups) as a factor. Secondary endpoints were analysed using analysis-of-covariance models with treatment group as a factor and baseline value of the relevant endpoint as covariate. Secondary endpoints that were proportions were analysed using Fisher's exact test. All statistical tests were two sided, and p values below 0.05 were considered significant. Analyses were performed using SAS V.9.3 software (Cary, North Carolina, USA).

## Results

**Figure 1:**
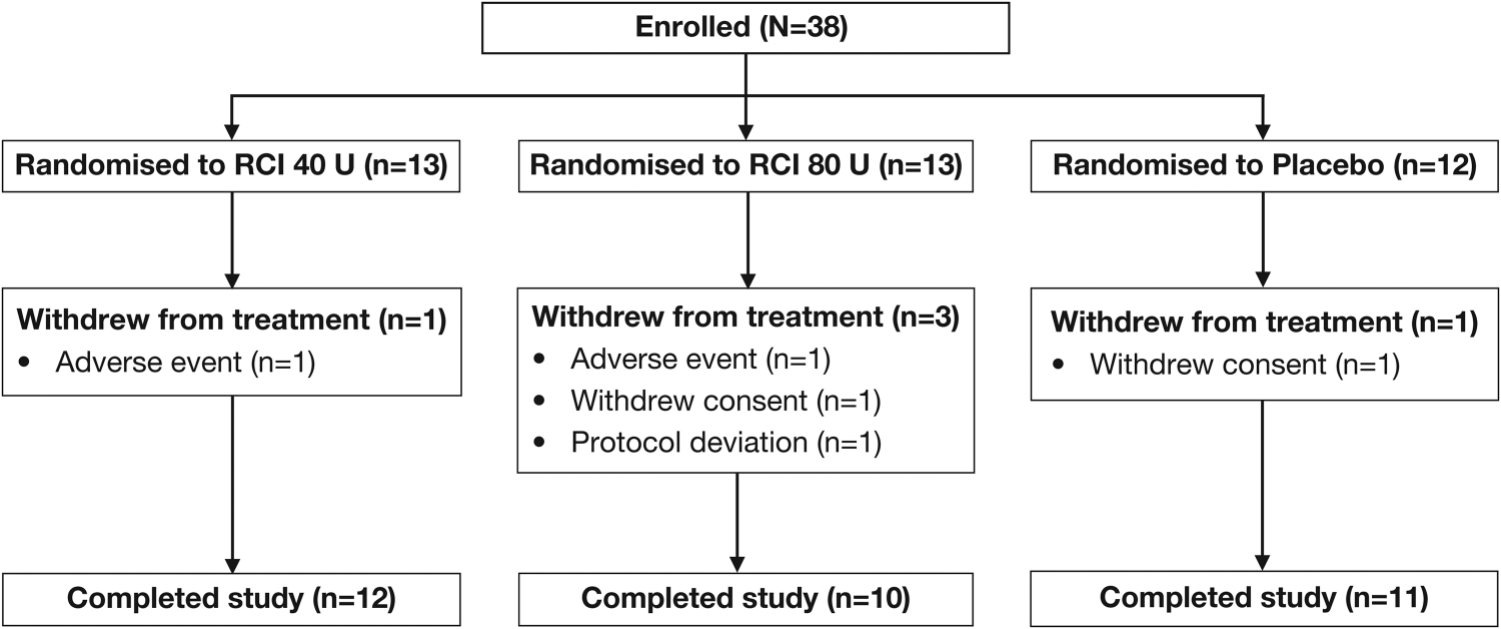
CONSORT diagram of study participants. RCI, repository corticotropin injection.

Thirty-eight patients were enrolled from January 2013 to December 2014, of whom 13 were randomised to receive RCI 40 U, 13 to RCI 80 U and 12 to placebo (figure 1). Five patients (13.2%) withdrew from the study during the double-blind period: one patient in each of the RCI groups was withdrawn because of AEs, two patients (one each in the combined placebo group and the RCI 80 U group) withdrew consent and were not included in the mITT population, and one patient receiving RCI 80 U was withdrawn because of a protocol deviation. The mITT population consisted of 36 patients.

Baseline demographic and disease characteristics of patients in the mITT population are summarised in table 1.

**Table 1:** Demographic characteristics and disease features

	Combined placebo (n=11)	Repository corticotropin injection 40 U (n=13)	Repository corticotropin injection 80 U (n=12)	Combined repository corticotropin injection (n=25)
Mean age (SD), years	39.1 (9.1)	42.6 (12.7)	43.2 (7.2)	42.9 (10.2)
Female, n (%)	10 (90.9)	12 (92.3)	12 (100)	24 (96.0)
*Race, n (%)*				
White	5 (45.5)	9 (69.2)	9 (75.0)	18 (72.0)
Black	6 (54.5)	3 (23.1)	3 (25.0)	6 (24.0)
hSLEDAI score, mean (SD)	9.8 (2.1)	8.7 (2.9)	11.3 (3.3)	10.0 (3.3)
Mean BILAG global score (SD)	15.4 (9.6)	13.1 (6.6)	18.6 (3.4)	15.7 (5.9)
Mean CLASI total activity score (SD)	6.1 (6.6)	5.9 (7.0)	7.0 (5.8)	6.4 (6.3)
Mean Tender and Swollen Joint Count (SD)	4.2 (4.8)	2.9 (3.4)	8.6 (6.8)*	5.6 (6.0)
Mean PGA (SD), mm	52.6 (12.5)	52.9 (14.3)	55.9 (11.9)	54.4 (13.0)
*PGA categories, n (%)*				
None	0	0	0	0
Mild	1 (9.1)	1 (7.7)	1 (8.3)	2 (8.0)
Moderate	8 (72.7)	10 (76.9)	9 (75.0)	19 (76.0)
Severe	2 (18.2)	2 (15.4)	2 (16.7)	4 (16.0)
Anti-dsDNA >5 IU/mL, n (%)	6 (54.5)	9 (69.2)	6 (50.0)	15 (60.0)
Complement C3 <LLN (0.87 g/L), n (%)	3 (27.3)	3 (23.1)	4 (33.3)	7 (28.0)
Complement C4 <LLN (0.19 g/L), n (%)	6 (54.5)	6 (46.2)	2 (16.7)	8 (32.0)
Mean prednisone (SD), mg/day	16.4 (8.1)	10.8 (2.6)†	9.2 (1.2)‡	10.0 (2.2)‡
Antimalarials, n (%)	8 (72.7)	11 (84.6)	7 (58.3)	18 (72.0)
Immunosuppressants, n (%)	6 (54.5)	4 (30.8)	2 (16.7)	6 (24.0)
Mycophenolate mofetil	4 (36.4)	2 (15.4)	1 (8.3)	3 (12.0)
Methotrexate	3 (27.3)	2 (15.4)	1 (8.3)	3 (12.0)
Azathioprine	0	1 (7.7)	2 (16.7)	3 (12.0)

*p=0.05 versus placebo; †p=0.007 versus placebo; ‡p=0.001 versus placebo.

PGA scores on a 100 mm visual analogue scale are categorised as follows: 0 point (none)=0 mm, 1 point (mild) >0–33.33 mm; 2 points (moderate) >33.33–66.67 mm and 3 points (severe) >66.67–100 mm.

BILAG, British Isles Lupus Assessment Group; CLASI, Cutaneous Lupus Erythematosus Disease Area and Severity Index; dsDNA, double-stranded DNA; hSLEDAI, hybrid SLE Disease Activity Index; LLN, lower limit of normal; PGA, Physician's Global Assessment.

Based on the hSLEDAI organ domains, the most common disease manifestations in the overall study population at baseline were arthritis (83.3%), alopecia (80.6%) and rash (75.0%) (see online supplementary file 2). Mean (SD) hSLEDAI scores were as follows: 9.8 (2.1), 8.7 (2.9) and 11.3 (3.3) in the combined placebo, RCI 40 U and RCI 80 U groups, respectively. Similar proportions of patients were receiving antimalarial therapies at baseline, but more patients in the placebo group were taking immunosuppressant therapy. The mean prednisone daily dose was 16.4 mg/day in the combined placebo group and 10.8 g/day and 9.2 g/day in the RCI 40 and 80 U groups, respectively.

10.1136/lupus-2016-000180.supp2 supplementary file

### Efficacy

**Figure 2:**
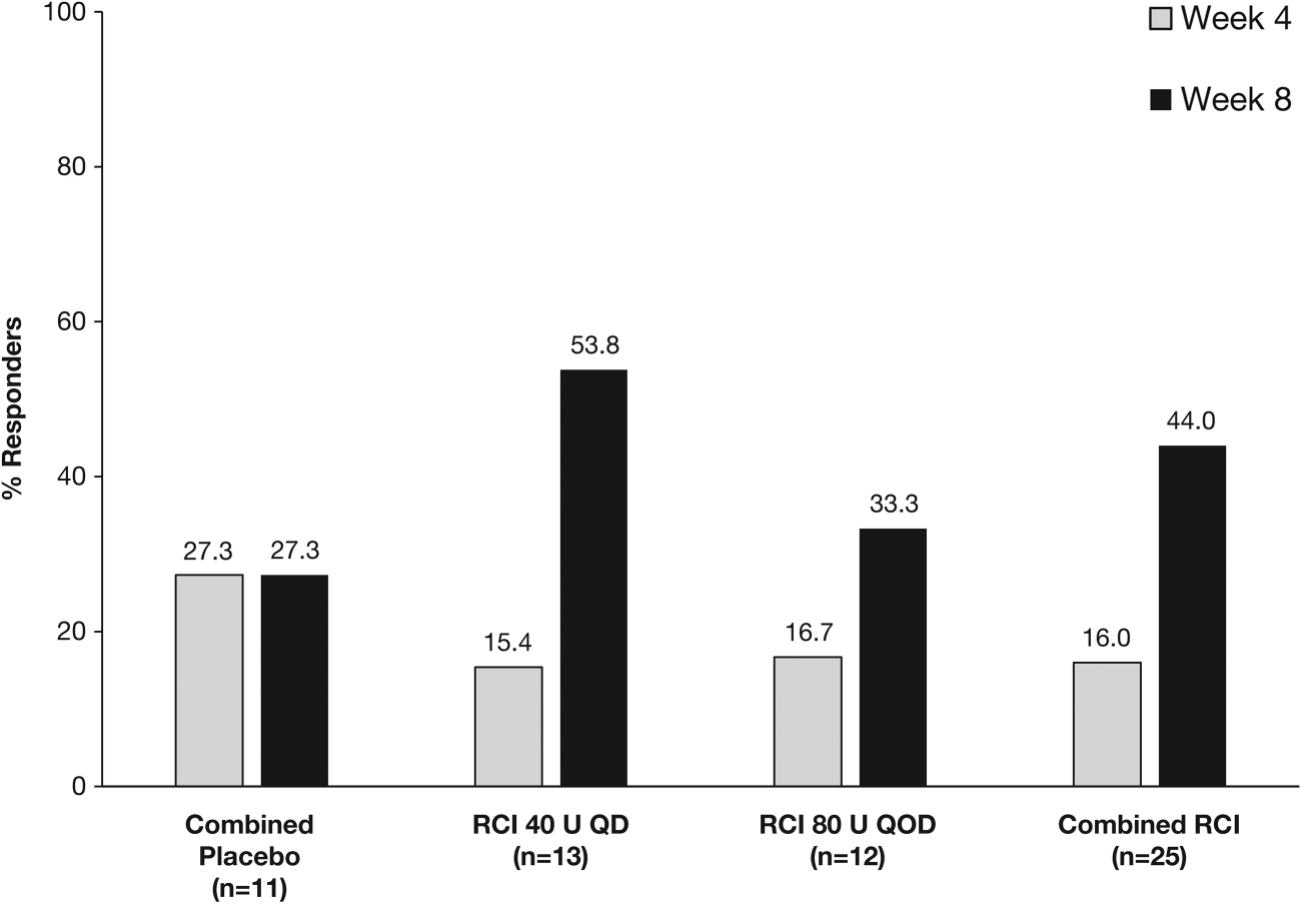
Proportion of patients showing a response to treatment at weeks 4 and 8. QD, once daily; QOD, once every other day; RCI, repository corticotropin injection.

**Figure 3:**
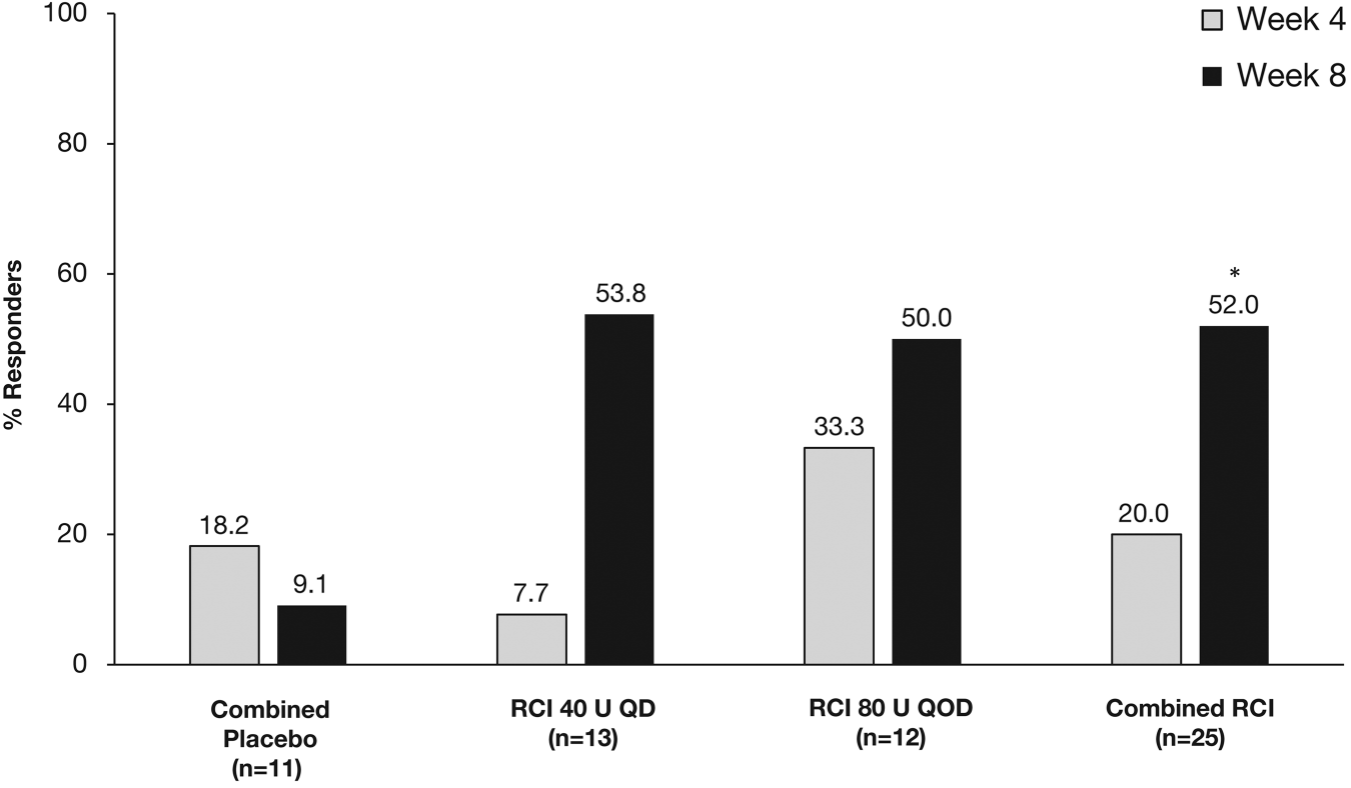
Response rate as defined by Systemic Lupus Erythematosus Responder Index at weeks 4 and 8. *p<0.05. QD, once daily; QOD, once every other day; RCI, repository corticotropin injection.

At week 4, there was no significant difference in the protocol-defined primary endpoint of response between the RCI and combined placebo groups (figure 2). At week 8, the proportion of patients defined as responders to treatment was numerically higher in both RCI groups than in the combined placebo group, but these differences did not reach statistical significance (figure 2). However, in post hoc analyses, the proportion of SRI responders was significantly higher in the combined RCI group than in the combined placebo group at week 8 (p=0.032; figure 3).

**Figure 4:**
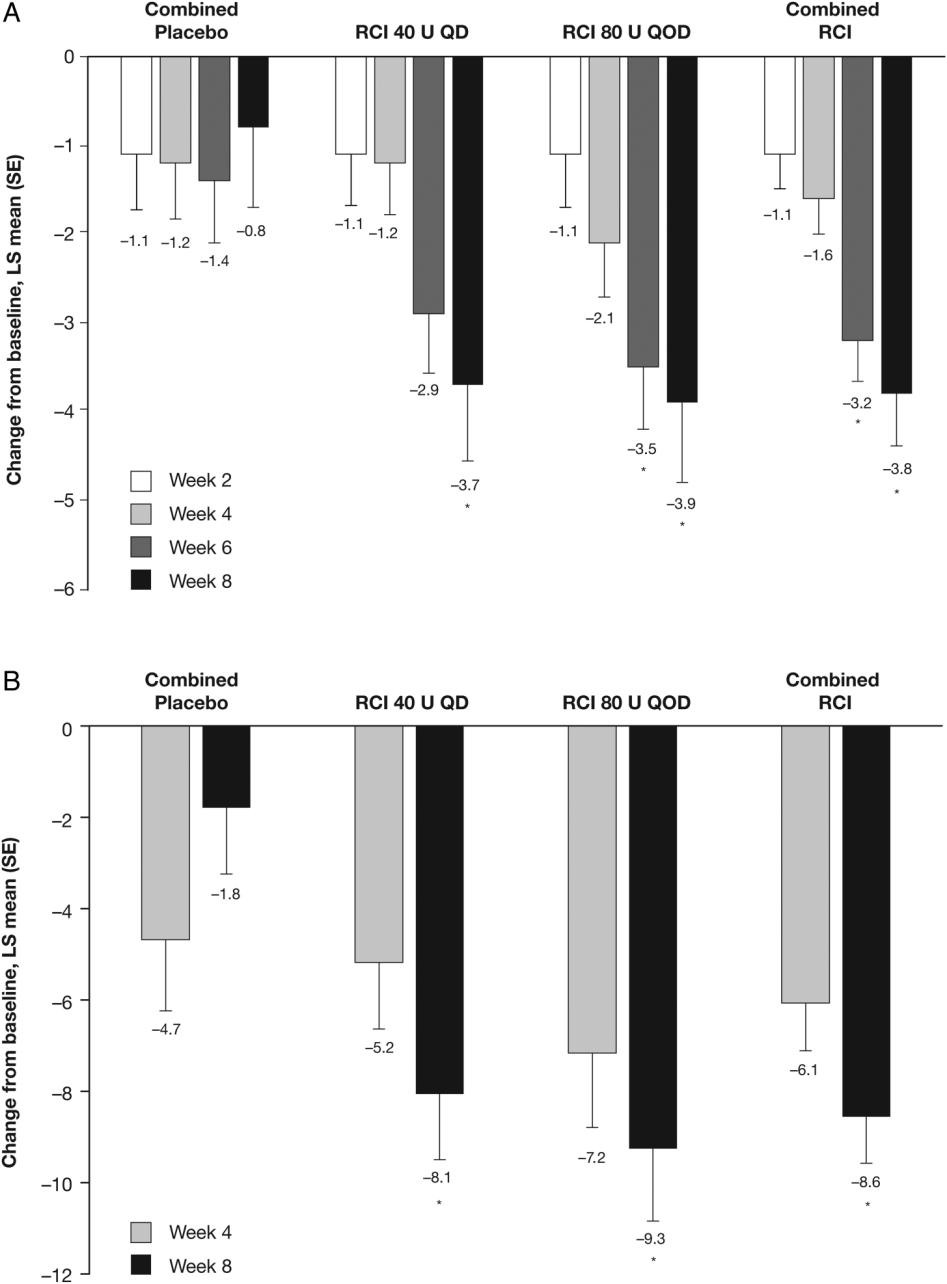
Least squares (LS) mean (SE) changes from baseline in (A) hybrid SLE Disease Activity Index scores at weeks 2, 4, 6 and 8, and (B) global British Isles Lupus Assessment Group scores at weeks 4 and 8. Values shown below error bars are LS means. *p<0.05. QD, once daily; QOD, once every other day; RCI, repository corticotropin injection.

**Figure 5:**
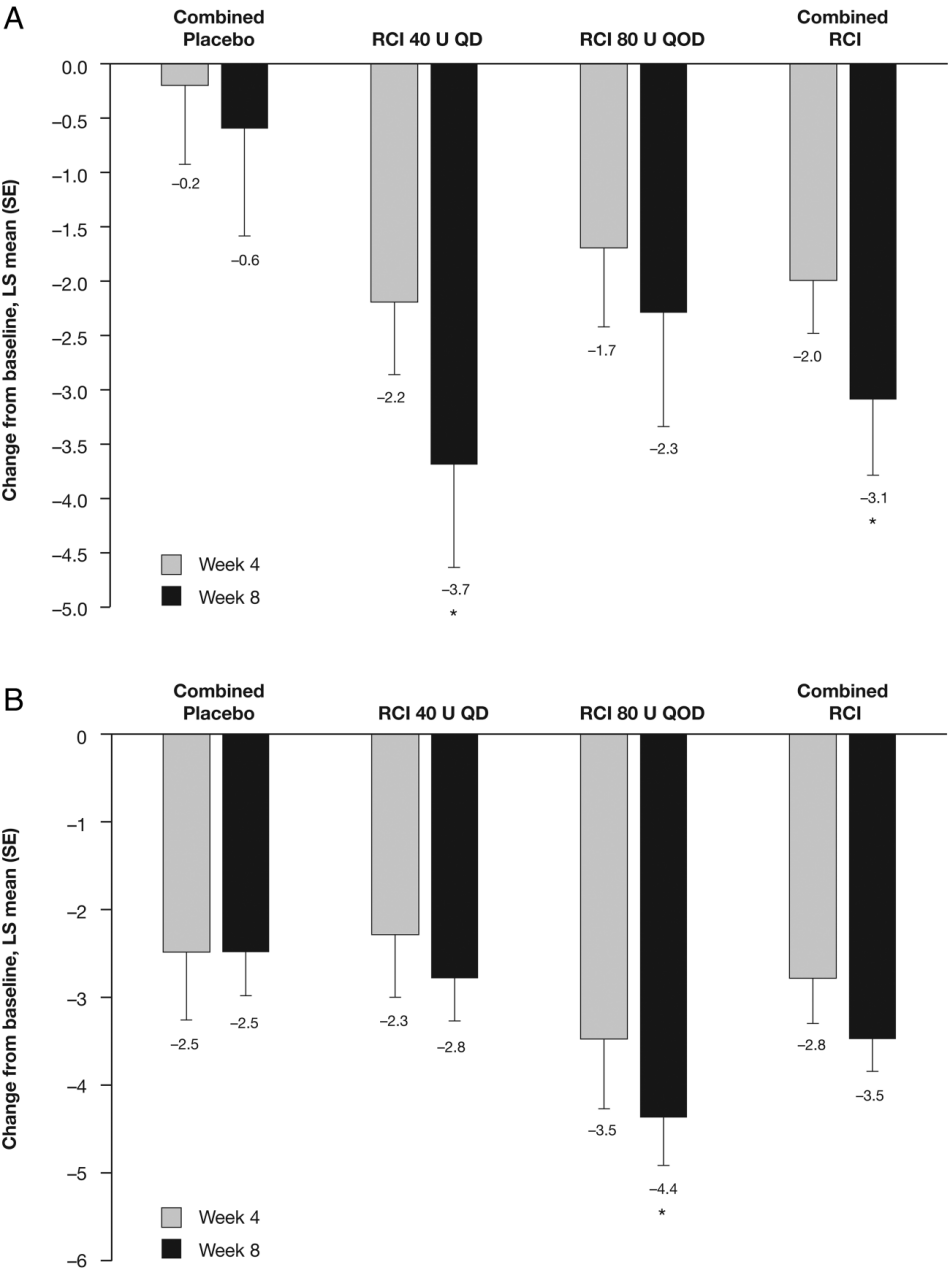
Least squares (LS) mean (SE) changes from baseline in (A) Cutaneous Lupus Erythematosus Disease Area and Severity Index activity scores and (B) Tender and Swollen Joint Count at weeks 4 and 8. Values shown below error bars are LS means. *p<0.05. QD, once daily; QOD, once every other day; RCI, repository corticotropin injection.

Although no statistically significant differences were noted between groups in change from baseline in total hSLEDAI scores at weeks 2 and 4, by week 6 the improvement from baseline in total hSLEDAI score was significantly greater in patients receiving RCI 80 U and in the combined RCI group compared with the combined placebo group (figure 4A); at week 8, significantly greater improvements in total hSLEDAI scores were seen in both RCI groups (40 U, p=0.026; 80 U, p=0.020) and in the combined RCI group (p=0.008) compared with the combined placebo group. Similarly, by week 8, improvements from baseline in total BILAG scores were significantly greater in both RCI dose groups (40 U, p=0.005; 80 U, p=0.002) and the combined RCI group (p=0.001) compared with the combined placebo group (figure 4B), and the proportion of patients with BILAG mucocutaneous or musculoskeletal domain score improvements was significantly higher in the RCI 80 U than combined placebo cohorts (83.3% vs 36.4%, p=0.036; see online supplementary file 3). Corresponding improvements in SLE skin manifestations were reflected by statistically significant improvements in CLASI activity scores in the RCI 40 U (p=0.030) and combined RCI (p=0.047) groups compared with the combined placebo group, at week 8. Similarly, at week 8, the Tender and Swollen Joint Count was statistically significantly improved from baseline in patients receiving RCI 80 U compared with the combined placebo group (figure 5B).

10.1136/lupus-2016-000180.supp3 supplementary file

**Figure 6:**
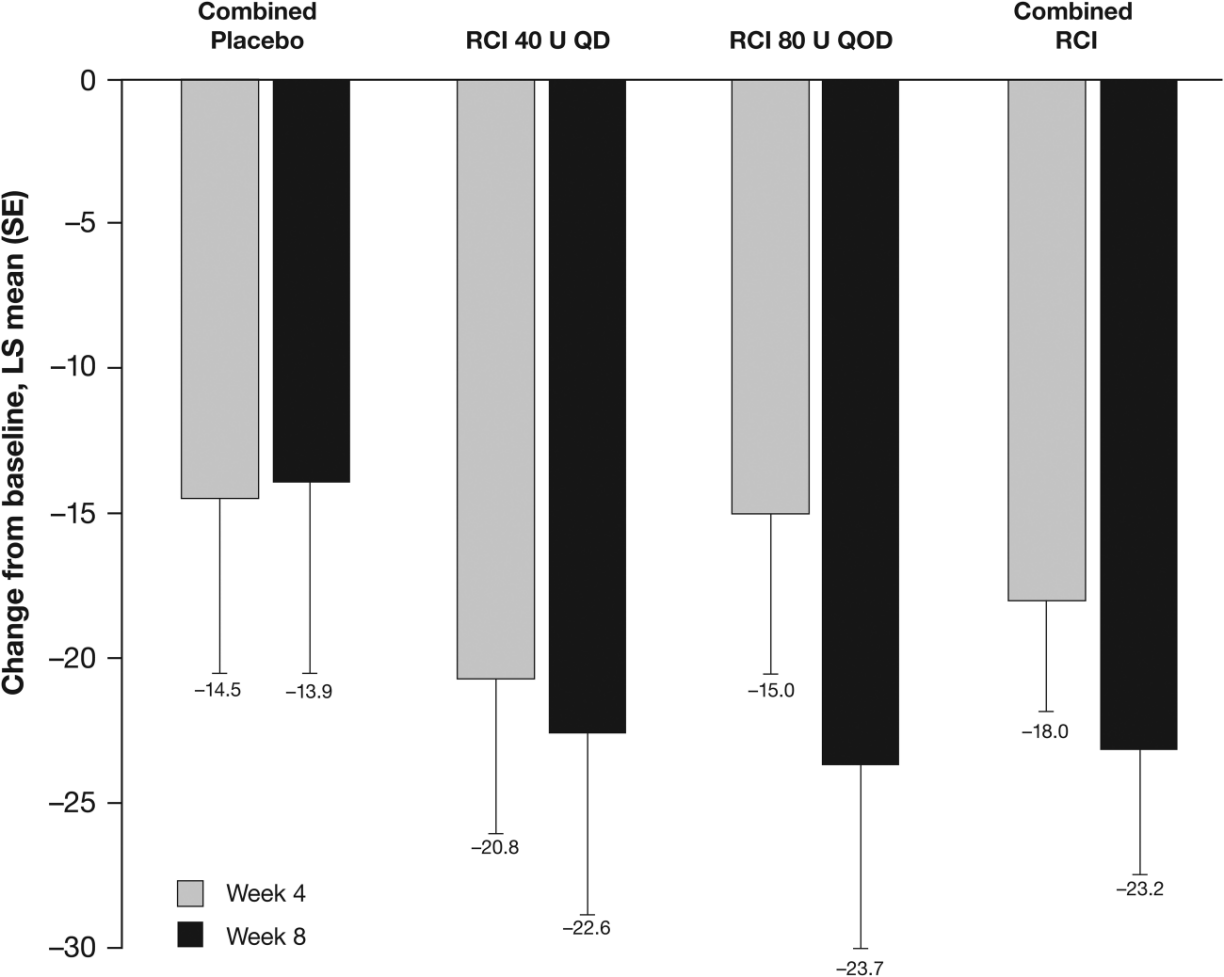
Least squares (LS) mean (SE) changes from baseline in Physician's Global Assessment scores at weeks 4 and 8. Values shown below error bars are LS means. QD, once daily; QOD, once every other day; RCI, repository corticotropin injection.

There were no statistically significant between-group differences in changes from baseline for PGA at either week 4 or 8, although PGA tended to improve more in RCI-treated patients as compared with placebo (figure 6). RCI 80 U was associated with a statistically significant improvement from baseline in aggregated SF-36 mental score at week 4, but this difference was not significant at week 8. There were no significant between-group differences in changes from baseline for aggregate SF-36 physical scores or KFSS at either time point (see online supplementary file 4). No notable changes in anti-dsDNA, C3 or C4 were seen during the 8-week study (data not shown).

10.1136/lupus-2016-000180.supp4 supplementary file

### Safety and tolerability

The overall incidences of treatment-emergent adverse events (TEAEs) and treatment-related TEAEs in the combined RCI and combined placebo groups were similar (see online supplementary file 5). The most commonly reported AE was weight gain, which occurred in seven patients (19.4%) with similar frequencies in each group. The overall incidence of infections was higher in the RCI groups (23.1% in each group) than in the combined placebo group (9.1%), but there were no other differences in AE profiles between the groups. The majority of TEAEs, including infections, were mild or moderate in severity.

10.1136/lupus-2016-000180.supp5 supplementary file

TEAEs leading to treatment discontinuation during the double-blind treatment period occurred in one patient (7.7%) receiving RCI 40 U and one patient (8.3%) receiving RCI 80 U. The patient in the RCI 40 U group experienced moderate chest discomfort and moderate gastro-oesophageal reflux disease, both of which were considered serious adverse events (SAEs) and related to study medication. The patient in the RCI 80 U group discontinued treatment because of a false-positive hepatitis C test result. This patient subsequently died from severe *Klebsiella* sepsis with multi-organ failure, which was considered unlikely to be related to study medication. A second patient in the RCI 80 U group was noted to have two SAEs, haemorrhagic ovarian cyst and viral infection, which were considered to be moderate in severity and unrelated to study medication, and did not lead to treatment discontinuation (see online supplementary file 5).

During weeks 1–4, three patients (two in the RCI 40 U group and one in the RCI 80 U group) had their RCI dose decreased based on tolerability. All three patients were also taking prednisone 10 mg daily. The events leading to dose reduction included moderate weight gain and mild increased tendency to bruise in the RCI 40 U group, and moderate irritability in the RCI 80 U group.

There were no clinically significant changes in physical examination findings, vital signs including blood pressure or clinical laboratory tests during the study.

## Discussion

The results of the double-blind, randomised phase of this pilot study provide contemporary controlled evidence to suggest that RCI may be a potential treatment alternative to improve disease activity for patients with SLE who have refractory rash and/or arthritis despite moderate-dose corticosteroid therapy. Although the primary endpoint was not met, the study did demonstrate improved disease activity in patients receiving RCI as compared with placebo as reflected by total hSLEDAI and BILAG scores. SLEDAI and BILAG are widely accepted SLE disease activity indices that are commonly used in lupus clinical trials.^24^ While the SLEDAI and BILAG scoring systems have been used extensively for clinical investigations in SLE, they have limitations. SLEDAI, for example, is unable to capture partial but potentially clinically important improvement in disease activity, and worsening of a pre-existing manifestation does not yield a change in score.^25^ The improvements in both global (hSLEDAI) and organ-specific (BILAG) disease activity measures in patients receiving RCI as compared with placebo strengthen the evidence that RCI reduces disease burden in this subpopulation of patients with SLE. Concordant improvements in skin or arthritic manifestations of disease as reflected by the CLASI activity score and Tender and Swollen Joint Count in RCI-treated patients provide yet further support of the efficacy of RCI in this SLE subpopulation. The CLASI scoring system provides a validated measure of cutaneous involvement that has been shown to be responsive to treatment-induced reductions in lupus skin lesions.^26^ Both CLASI and the Tender and Swollen Joint Count assess cutaneous and musculoskeletal manifestations of SLE at a specific point in time, and have been used in other investigational SLE trials to verify measures of disease activity in these organ systems captured by SLEDAI and BILAG.^19^

Significant response to RCI was not reflected by the PGA, a subjective assessment describing global disease activity at a single point in time.^17^ However, post hoc analysis demonstrated that, by week 8, over half of RCI-treated patients were responders, as measured by a well-established composite responder index, the SRI,^22^ compared with fewer than 20% of patients receiving placebo.

Statistically significant differences in changes from baseline for QoL measures (SF-36 and KFSS) were also not detected with consistency between RCI-treated and placebo-treated patients over the 8-week study period. This might reflect a lack of impact of RCI on QoL measures due to the small sample, the short duration of therapy or a true lack of benefit of the intervention. Analysis of serial SF-36 scores obtained over an average of 8 years in a large cohort of patients with established SLE revealed that the SF-36 aggregate, mental and physical component scores did not change appreciably over time.^27^ The authors concluded that these measures may not be sensitive to changes in disease activity or damage. Although Furie *et al* demonstrated significant improvements in fatigue and QoL metrics in a post hoc analysis of the phase 3 belimumab data set, the comparison in their analysis was between SRI responders and non-responders, irrespective of treatment assignment.^28^

The response to treatment with RCI in the SLE population studied was seen as early as week 6 for improvements from baseline in total hSLEDAI score, and by week 8 significant improvements were seen in most objective and composite measures of disease activity (total BILAG, improvement in BILAG A and B scores, CLASI activity and Tender and Swollen Joint Count). An alternative approach to the treatment of active SLE that has been evaluated in clinical trials^29^ ^30^ is the use of B-cell inhibitors such as belimumab. In the BLISS-52 study, response rates as measured by the SRI were significantly higher with belimumab than with placebo.^29^ However, clinical improvement was not seen until 16 weeks, whereas in the current study, improvements in disease activity occurred as early as 6–8 weeks after the start of treatment with RCI. Although the novel composite primary endpoint for the study was negative, the statistically significant improvements seen for multiple other concordant measures of SLE disease activity achieved by week 8 are notable given the small sample size. Taken together, these controlled data suggest that RCI may be an effective therapy for SLE, although the applicability of these data to a wider SLE patient population will need to be tested in future controlled trials.

RCI was well tolerated in this study. The overall incidence of AEs was comparable to the placebo group, and the majority of AEs were mild or moderate. The higher incidence of infections in patients receiving RCI may be related to the steroidogenic effects of ACTH.^8^

Strengths of this study include the use of well-validated outcome measures that evaluate articular, systemic and cutaneous manifestations of SLE; objective assessments of global and organ-specific disease activity with varying recall periods; physician-reported and patient-reported subjective outcome measures; and the inclusion of patients with persistent disease despite moderate doses of corticosteroids, a more difficult-to-treat subset of patients.

The study has a number of limitations, however, notably that no formal sample size calculations to achieve statistical power were performed. Because of the small sample size, patients were not stratified based on disease severity nor background therapies, and potential imbalances in either of these parameters might have influenced response to treatment. In addition, the primary endpoint was a responder index that has not been reported in other clinical investigations of SLE therapies. Potential explanations for the discrepancy between response assessed by the primary endpoint and the more conventional SLE disease activity indices include the small sample size, limitation of the novel responder index to reflect improvement in SLEDAI domains other than rash and arthritis, and a more stringent definition for BILAG worsening (no worsening in any organ system by BILAG) as compared with previously validated composite response measures such as the SRI and BILAG-based Combined Lupus Assessment (no new BILAG A or no more than one new BILAG B domain score).

In conclusion, the data from this controlled pilot study suggest that RCI may be effective in reducing disease activity in patients with steroid-dependent SLE involving skin and/or joints, and will inform the design of future clinical investigations of RCI in SLE.
