# Supplementary material for: Efficacy and tolerability of repository corticotropin injection in patients with persistently active SLE: results of a phase 4, randomised, controlled pilot study
Source: Lupus Sci Med. 2016 Oct 21;3(1):e000180. doi: 10.1136/lupus-2016-000180 (PMC5133412; doi:10.1136/lupus-2016-000180)
Supplement: supplementary file [file lupus-2016-000180supp1.pdf]

Online supplementary file 1 US study sites and principal investigators

Principal Investigator	Site	City, State
Emily Jane Herron Box	Box Arthritis & Rheumatology of the Carolinas, PLLC	Charlotte, North Carolina
Elizabeth M Bretton	Albuquerque Clinical Trials, Inc.	Albuquerque, New Mexico
Jill Pamela Buyon	NYU Center for Musculoskeletal Care	New York, New York
Patricia Cagnoli	Beals Institute PC	Lansing, Michigan
Gregory Emkey	Pennsylvania Regional Center for Arthritis & Osteoporosis Research	Wyomissing, Pennsylvania
Benidecto Fernandez	Lakes Research, LLC	Miami Lakes, Florida
Justus J Fiechtner	Justus J. Fiechtner, MD, MPH	Lansing, Michigan
Beata Joanna Filip-Majewski	NEA Baptist Clinic	Jonesboro, Arkansas
Pamela G Freeman	Rheumatology Associates of Central Florida, PA	Orlando, Florida
Richard Furie	North Shore-LIJ Health System	Great Neck, New York
Gerald Ho	Arthritis and Osteoporosis Medical Center, Inc.	La Palma, California
Eric C Lee	Inland Rheumatology Clinical Trials, Inc.	Upland, California
Robert William Levin	Clinical Research of West Florida, Inc.	Clearwater, Florida
Stephen M Lindsey	Ochsner Clinic Foundation – Baton Rouge	Baton Rouge, Louisiana
Kimberly McIlwain Smith	McIlwain Medical Group, PA	Tampa, Florida
Nathaniel Neal	Valerius Medical Group and Research Center of Greater Long Beach, Inc.	Long Beach, California
Nancy Olsen	Penn State Milton S. Hershey Medical Center	Hershey, Pennsylvania
Samuel B Pegram	Rheumatic Disease Clinical Research Center	Houston, Texas
Nicholas R Straniero	Memorial Medical Group Rheumatology Center	Granger, Indiana
Philip A Waller	Accurate Clinical Research, Inc.	Houston, Texas
