# Supplementary material for: Efficacy and tolerability of repository corticotropin injection in patients with persistently active SLE: results of a phase 4, randomised, controlled pilot study
Source: Lupus Sci Med. 2016 Oct 21;3(1):e000180. doi: 10.1136/lupus-2016-000180 (PMC5133412; doi:10.1136/lupus-2016-000180)
Supplement: supplementary file [file lupus-2016-000180supp2.pdf]

Online supplementary file 2 Summary of SLEDAI manifestation by treatment group at baseline (mITT population)

Item no.	SLEDAI score	SLEDAI descriptor	Combined Placebo (n=11)	RCI 40 U QD (n=13)	RCI 80 U QOD (n=12)	Combined RCI (n=25)
01	8	Seizure	0	0	0	0
02	8	Psychosis	0	0	0	0
03	8	Organic brain syndrome	0	0	0	0
04	8	Visual disturbance	0	0	0	0
05	8	Cranial nerve disorder	0	0	0	0
06	8	Lupus headache	0	0	0	0
07	8	Cerebrovascular accident	0	0	0	0
08	8	Vasculitis	1 (9.1)	0	1 (8.3)	1 (4.0)
09	4	Arthritis	8 (72.7)	10 (76.9)	12 (100.0)	22 (88.0)
10	4	Myositis	1 (9.1)	0	0	0
11	4	Urinary casts	0	0	0	0
12	4	Haematuria	0	0	0	0
13	4	Proteinuria	0	0	0	0
14	4	Pyuria	0	0	0	0
15	2	Rash	8 (72.7)	8 (61.5)	11 (91.7)	19 (76.0)
16	2	Alopecia	9 (81.8)	10 (76.9)	10 (83.3)	20 (80.0)
17	2	Mucosal ulcers	4 (36.4)	4 (30.8)	8 (66.7)	12 (48.0)
18	2	Pleurisy	1 (9.1)	1 (7.7)	2 (16.7)	3 (12.0)
19	2	Pericarditis	0	0	0	0
20	2	Low complement	7 (63.6)	7 (53.8)	4 (33.3)	11 (44.0)
21	2	Increased DNA binding	3 (27.3)	6 (46.2)	5 (41.7)	11 (44.0)
22	1	Fever	0	0	0	0
23	1	Thrombocytopenia	0	0	0	0
24	1	Leukopenia	0	1 (7.7)	0	1 (4.0)

Values presented as n (%) of patients.

mITT, modified intention-to-treat; QD, once daily; QOD, once every other day; RCI, Repository Corticotropin Injection; SLEDAI, Systemic Lupus Erythematosus Disease Activity Index.
