# Supplementary material for: Efficacy and tolerability of repository corticotropin injection in patients with persistently active SLE: results of a phase 4, randomised, controlled pilot study
Source: Lupus Sci Med. 2016 Oct 21;3(1):e000180. doi: 10.1136/lupus-2016-000180 (PMC5133412; doi:10.1136/lupus-2016-000180)
Supplement: supplementary file [file lupus-2016-000180supp3.pdf]

Online supplementary file 3 Proportion of patients achieving improvement in BILAG category for mucocutaneous or musculoskeletal body systems (mITT population)

Time point	Statistics	Combined Placebo (n=11)	RCI 40 U QD (n=13)	RCI 80 U QOD (n=12)	Combined RCI (n=25)
Week 4	Improvement*	3 (27.3)	5 (38.5)	8 (66.7)	13 (52.0)
	No improvement	8 (72.7)	8 (61.5)	4 (33.3)	12 (48.0)
	p-value†	–	0.679	0.100	0.277
Week 8	Improvement*	4 (36.4)	7 (53.8)	10 (83.3)	17 (68.0)
	No improvement	7 (63.6)	6 (46.2)	2 (16.7)	8 (32.0)
	p-value†	–	0.444	0.036‡	0.141

Values presented as n (%) of patients.
*Improvement was defined as category A at baseline to B/C/D at a specific post-baseline visit, or B at baseline to C/D at the specific visit. Patients with missing data were classified as 'No improvement'.
†p-value based on Fisher's exact test comparing the combined placebo with each RCI group.
‡p<0.05.
BILAG, British Isles Lupus Assessment Group; mITT, modified intention-to-treat; RCI, Repository Corticotropin Injection.
