# Supplementary material for: Efficacy and tolerability of repository corticotropin injection in patients with persistently active SLE: results of a phase 4, randomised, controlled pilot study
Source: Lupus Sci Med. 2016 Oct 21;3(1):e000180. doi: 10.1136/lupus-2016-000180 (PMC5133412; doi:10.1136/lupus-2016-000180)
Supplement: supplementary file [file lupus-2016-000180supp4.pdf]

Online supplementary file 4 Changes in SF-36 and Krupp Fatigue Severity scores from baseline to weeks 4 and 8 (mITT population)

	**Change from baseline, LS mean (SE)**		
	**Combined Placebo (n=11)**	**RCI 40 U QD (n=13)**	**RCI 80 U QOD (n=12)**
SF-36 aggregated mental scores			
Week 4	–0.37 (2.65)	–3.30 (2.47)	8.90 (2.70)*
Week 8	1.50 (3.21)	–1.75 (3.15)	7.27 (3.43)
SF-36 aggregated physical scores			
Week 4	3.11 (1.80)	4.27 (1.64)	2.20 (1.79)
Week 8	1.42 (1.94)	4.17 (1.86)	1.65 (2.03)
Krupp Fatigue Severity score			
Week 4	–0.16 (0.41)	–0.22 (0.38)	–0.33 (0.42)
Week 8	0.01 (0.37)	–0.50 (0.35)	–0.31 (0.39)

*p=0.02.

LS, least squares; mITT, modified intention-to-treat; RCI, Repository Corticotropin Injection; SE, standard error; SF-36, Medical Outcomes Survey Short Form-36.
