# Supplementary material for: Efficacy and tolerability of repository corticotropin injection in patients with persistently active SLE: results of a phase 4, randomised, controlled pilot study
Source: Lupus Sci Med. 2016 Oct 21;3(1):e000180. doi: 10.1136/lupus-2016-000180 (PMC5133412; doi:10.1136/lupus-2016-000180)
Supplement: supplementary file [file lupus-2016-000180supp5.pdf]

Online supplementary file 5 Summary of treatment-emergent adverse events

**Category**	**Combined Placebo (n=11)**	**RCI 40 U QD (n=13)**	**RCI 80 U QOD (n=12)**	**Combined RCI (n=25)**
Any TEAE	9 (81.8)	12 (92.3)	7 (58.3)	19 (76.0)
Severe TEAEs	1 (9.1)	0	1 (8.3)	1 (4.0)
Treatment-related TEAEs	4 (36.4)	7 (53.8)	4 (33.3)	11 (44.0)
TEAEs leading to withdrawal from study*	0	3 (23.1)	1 (8.3)	4 (16.0)
Serious TEAEs	0	2 (15.4)	1 (8.3)	3 (12.0)
TEAEs resulting in death	0	0	1 (8.3)	1 (4.0)
**AEs occurring in >1 patient in the combined RCI group**				
Increased weight	2 (18.2)	3 (23.1)	2 (16.7)	5 (20.0)
Abdominal pain	0	2 (15.4)	0	2 (8.0)
Back pain	0	1 (7.7)	1 (8.3)	2 (8.0)
Diarrhoea	0	1 (7.7)	1 (8.3)	2 (8.0)
Fatigue	0	0	2 (16.7)	2 (8.0)
Fluid retention	0	1 (7.7)	1 (8.3)	2 (8.0)
Irritability	0	1 (7.7)	1 (8.3)	2 (8.0)
Mood swings	0	2 (15.4)	0	2 (8.0)
Oropharyngeal pain	0	0	2 (16.7)	2 (8.0)

Values presented as n (%) of patients.

*Two of these withdrawals occurred during the open-label phase of the study.

AE, adverse event; RCI, Repository Corticotropin Injection; TEAE, treatment-emergent adverse event.
